# Draft Genome Sequence of Mycolicibacterium smegmatis VKM Ac-1171 Contains Full Set of Sterol Catabolic Genes

**DOI:** 10.1128/mra.00772-22

**Published:** 2022-11-10

**Authors:** Dmitry V. Dovbnya, Eugeny Y. Bragin, Tanya V. Ivashina, Marina V. Donova

**Affiliations:** a G. K. Skryabin Institute of Biochemistry and Physiology of Microorganisms, Pushchino Center for Biological Research, Russian Academy of Sciences, Pushchino, Russia; Montana State University

## Abstract

*Mycolicibacterium smegmatis* VKM Ac-1171 is a saprotrophic bacterium that was isolated several decades ago and is deposited in microbial collections around the world. We report here a draft genome sequence of the strain. Annotation of the genome revealed the presence of a complete set of genes related to the sterol catabolic pathway.

## ANNOUNCEMENT

*Mycolicibacterium smegmatis* VKM Ac-1171 (NCIMB 8548, CCM 2067) was originally deposited in ATCC decades ago as Mycobacterium butyricum 362 and later reidentified as Mycobacterium smegmatis (1). Recently, Mycobacterium smegmatis and closely related fast-growing species have been reclassified as *Mycolicibacterium* based on their phylogenomic differences from the “tuberculosis-like” clade (2, 3). The strain has previously been used in various studies as a nonpathogenic model microorganism (4–9), to validate the DNA isolation method (10), but its genome sequence was hitherto unreported. Here, we present a draft genome of M. smegmatis VKM Ac-1171.

The strain was obtained from the All-Russian Collection of Microorganisms VKM (http://www.vkm.ru) and cultured aerobically at 37°C to early stationary phase in MYCB broth (11) supplemented with 15 g/L Tween 80 and 15 g/L glycine.

Genomic DNA was extracted as described (12) with modifications. Briefly, cells from 10 mL broth were subjected to sequential treatment with lysozyme (20 min, 37°C), SDS, proteinase K (1.0 h, 56°C), and RNase A (30 min, 37°C). Then, the DNA was purified with phenol-chloroform.

The Illumina sequencing library construction was made by KAPA DNA library preparation kit for Illumina and KAPA dual-indexed adapter kit (Kapa Biosystems). Genome sequencing was performed by Illumina HiSeq 2000 with HiSeq SBS kit v3. For adapter and quality trimming, Trimmomatic 0.39 (13) with the settings ILLUMINACLIP:TruSeq3-PE:2:30:10:2, LEADING:3, TRAILING:3, MINLEN:50, and a self-written program in Perl language (https://github.com/BraginE/bioinfo) were applied. *De novo* genome assembly was made with the Ray 2.3.1 program (14); the k-mer length was 31. Genome was annotated with Prokaryotic Genome Annotation Pipeline (PGAP) (15). For average nucleotide identity (ANI), the ANI calculator (16) was applied. Default parameters were used for all software unless mentioned otherwise.

Sequencing resulted in 19,143,437 paired-end reads (2 × 100). The genome assembly generated 96 contigs with 7,600,730-bp total length (genome coverage, 44×; *N*_50_ length, 199,025 bp; GC content, 67.5%).

Among M. smegmatis strains with known genome sequences, Ac-1171 showed the highest similarity to M. smegmatis Nishi, whereas the ANI value between Ac-1171 and M. smegmatis mc^2^ 155 was lower (Table 1).

**TABLE 1:** Average nucleotide identities between genomes of VKM Ac-1171 and other Mycolicibacterium smegmatis strains

Strain	GenBank accession no.	Genome length (bp)	ANI value
M. smegmatis mc^2^ 155	CP009494	6,988,269	98.94
M. smegmatis strain Rabinowitchi	CP080272	7,061,747	99.01
M. smegmatis strain Nishi	CP080273	7,010,278	99.09
M. smegmatis strain Jucho	CP080274	6,895,172	98.95

The size of the Ac-1171 genome is approximately 600,000 bp bigger than the genomes of other M. smegmatis strains. The Ac-1171 genome contains 7,163 protein-coding genes, 57 RNA-coding genes (2, 2, 2, 48 and 3 genes coding for 5S rRNA, 16S rRNA, 23S rRNA, tRNA, and noncoding RNA, respectively), and 167 pseudogenes. The strain Ac-1171 possesses a complete set of key genes of steroid catabolism, thus suggesting the ability for the full sterol degradation.

Modification of sterol catabolic pathways in some species of *Mycolicibacterium*, such as M. neoaurum (17), M. fortuitum (18), and M. smegmatis mc^2^ 155 (19), has become the basis for production of pharmaceutical steroid precursors. The strain M. smegmatis VKM Ac-1171 is promising for the engineering of novel microbial producers for steroid biotechnology.

### Data availability.

The genome sequences have been deposited in NCBI GenBank database under accession number JAMZOD000000000. The BioSample and BioProject accession numbers are SAMN28113943 and PRJNA835822, respectively. The draft genome raw data are available in the Sequence Read Archive (SRA) under accession number SRR19122810.
